# The Mucosal and Serological Immune Responses to the Novel Coronavirus (SARS-CoV-2) Vaccines

**DOI:** 10.3389/fimmu.2021.744887

**Published:** 2021-10-12

**Authors:** Renee W. Y. Chan, Shaojun Liu, Jonathan Y. Cheung, Joseph G. S. Tsun, Kate C. Chan, Kathy Y. Y. Chan, Genevieve P. G. Fung, Albert M. Li, Hugh Simon Lam

**Affiliations:** ^1^ Department of Paediatrics, Faculty of Medicine, The Chinese University of Hong Kong, Shatin, Hong Kong, SAR China; ^2^ Laboratory for Paediatric Respiratory Research, Li Ka Shing Institute of Health Sciences, Faculty of Medicine, The Chinese University of Hong Kong, Shatin, Hong Kong, SAR China; ^3^ The Chinese University of Hong Kong–University Medical Center Utrecht Joint Research Laboratory of Respiratory Virus and Immunobiology, Department of Paediatrics, Faculty of Medicine, The Chinese University of Hong Kong, Shatin, Hong Kong, SAR China; ^4^ Hong Kong Hub of Paediatric Excellence, The Chinese University of Hong Kong, Kowloon Bay, Hong Kong, SAR China

**Keywords:** mucosal immunity, nasal epithelial lining fluid, immunoglobulin A, immunoglobulin G, serological immunity, mRNA vaccine, inactivated virus vaccine, SARS-CoV-2

## Abstract

**Background:**

Although the serological antibody responses induced by SARS-CoV-2 vaccines are well characterized, little is known about their ability to elicit mucosal immunity.

**Objectives:**

This study aims to examine and compare the mucosal and systemic responses of recipients of two different vaccination platforms: mRNA (Comirnaty) and inactivated virus (CoronaVac).

**Methods:**

Serial blood and nasal epithelial lining fluid (NELF) samples were collected from the recipients of either Comirnaty or CoronaVac. The plasma and NELF immunoglobulins A and G (IgA and IgG) specific to SARS-CoV-2 S1 protein (S1) and their neutralization effects were quantified.

**Results:**

Comirnaty induced nasal S1-specific immunoglobulin responses, which were evident as early as 14 ± 2 days after the first dose. In 64% of the subjects, the neutralizing effects of NELF persisted for at least 50 days. Moreover, 85% of Comirnaty recipients exhibited S1-specific IgA and IgG responses in plasma by 14 ± 2 days after the first dose. By 7 ± 2 days after the booster, all plasma samples possessed S1-specific IgA and IgG responses and were neutralizing. The induction of S1-specific plasma antibodies by CoronaVac was IgG dominant, and 83% of the subjects possessed S1-specific IgG by 7 ± 2 days after the booster, with neutralizing effects.

**Conclusion:**

Comirnaty induces S1-specific IgA and IgG responses with neutralizing activity in the nasal mucosa; a similar response is not seen with CoronaVac.

**Clinical Implication:**

The presence of a nasal response with mRNA vaccine may provide additional protection compared with inactivated virus vaccine. However, whether such widespread immunological response may produce inadvertent adverse effects in other tissues warrants further investigation.

## Introduction

Severe acute respiratory syndrome coronavirus 2 (SARS-CoV-2) vaccines are among the most important measures against the coronavirus disease 2019 (COVID-19) pandemic, a public health threat that has resulted in a global death toll of over 4 million (1). Among the vaccines currently authorized by the World Health Organization (WHO), CoronaVac by Sinovac Biotech, an inactivated virus vaccine, and BNT162b2 (a.k.a. Comirnaty) from Pfizer-BioNTech, a messenger RNA (mRNA) vaccine encoding viral spike (S) protein (2), have been approved for emergency use in Hong Kong. Both vaccines have good safety records with low prevalence of serious adverse events (3–5). CoronaVac has been shown to prevent symptomatic COVID-19 in 51% of vaccinated healthcare workers and exhibits an efficacy of 100% in preventing severe COVID-19 in Brazil (6), while a recent study in Chile carried out from February to May 2021 indicates adjusted vaccine effectiveness of 65.9% for the prevention of infection, 90.3% for the prevention of ICU admission, and 86.3% for the prevention of COVID-19-related death (7). Comirnaty was reported to be 95% effective in preventing symptomatic COVID-19, with low incidence of serious adverse events (8). By 14 days after the CoronaVac booster, an earlier preprint reported that 95.6 and 95.7% of the recipients aged 18–50 years exhibited plasma spike protein 1-receptor binding domain (S1-RBD)-specific IgG and neutralizing antibodies, respectively (8). In contrast, two recent publications suggested that the recipients of this inactivated vaccine had low antibody concentrations after the first dose, rising to moderate concentrations after the second dose (9, 10). In comparison, 100% of Comirnaty recipients exhibited plasma S1-RBD-specific IgG by 21 days after the priming dose (11).

Comirnaty and Moderna elicit neutralizing antibody (NAb) responses that target the RBD epitopes in the same manner as natural infections (12). Albeit at much lower titers than IgG levels, the mRNA vaccines also induce IgM and IgA responses against S-protein and RBD in plasma samples (12). As detectable IgM levels after vaccinations are often significantly lower and less sustained when compared to IgA and IgG levels, IgM is suspected of having lesser importance in virus neutralization *in vivo* (12, 13). On the other hand, plasma S1-RBD IgA from COVID-19 patients has been found to have more potent neutralization potential than paired IgG (14). SARS-CoV-2 IgA can be sustainably elevated in serum or plasma samples for over 2 months after Comirnaty vaccination (13). Thus, the importance of systemic SARS-CoV-2 IgA in vaccine-induced immunity against COVID-19 warrants further validation (13, 15).

Secretory IgA (SIgA), being the most abundant immunoglobulin expressed on mucosal surfaces, often serves as the first line of defense against infections (14), and the circulating monomeric IgA cannot be readily transported into mucosal secretions, suggesting the likelihood of distinct systemic and mucosal responses to SARS-CoV-2 (14). A study has reported that mRNA vaccines induce detectable levels of salivary S1-RBD IgA and IgG; however, the capacity for viral neutralization was unknown (16). Determining whether the current vaccines induce antibody responses in the mucosa and if found so, the duration and sustainability of any such response can provide invaluable insight into the mechanisms of these vaccines and how best to utilize them.

With our recent work using nasal strips to collect nasal epithelial lining fluid (NELF) in SARS-CoV-2-infected children and adults, we have developed a method for SARS-CoV-2 nucleoprotein gene detection (17) that can be adapted for mucosal antibody quantification. We hypothesize that, in addition to systemic immune responses, SARS-CoV-2 vaccines can also induce SARS-CoV-2-specific antibodies in mucosal surfaces. Our objective was to compare the serological and mucosal immune responses after vaccination with CoronaVac and Comirnaty, with a particular focus on S1-specific immunoglobulins and neutralization capacity in plasma and NELF.

## Methods

### Subject Recruitment and Clinical Sample Collection Regime

**Figure 1:**
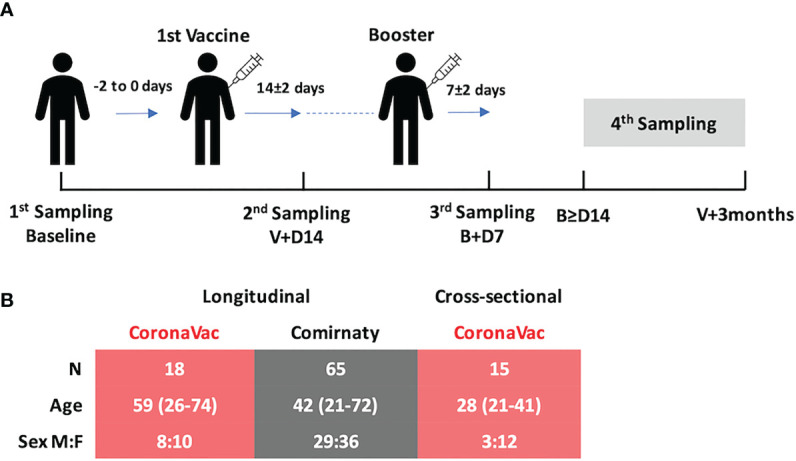
**(A)** Study design and demographics. There were three standard sampling time points and one extended sampling time point (fourth sampling) of biological sample collection: (i) 0 to 2 days before the first vaccination (baseline), (ii) 14 ± 2 days after the first vaccination (V+D14), (iii) 7 ± 2 days after the booster (B+D7), and (iv) any day between 14 days after the booster and before 3 months after the first vaccination. **(B)** Subjects vaccinated with CoronaVac (*n* = 18, pink table) and Comirnaty (*n* = 65, gray table) were recruited and followed longitudinally. There was a significant difference in their age distributions (*p* = 0.0061, Mann–Whitney test, two-tailed), and so 15 extra subjects vaccinated with CoronaVac were recruited to enrich the data for the fourth time point.

The subjects were individuals aged ≥18 years who were scheduled to receive the SARS-CoV-2 vaccine. Subject demographics, medical history, drug history, and any adverse effects after vaccination were recorded. NELF from both nares and plasma from 3 ml of peripheral blood was collected from the subjects at four time points: the 48-h period before the day of vaccination, at 14 ± 2 days after the first dose, at 7 ± 2 days post-booster (**Figure 1A**), and any day between 14 ± 2 days post-booster and before 3 months after the first vaccination dose. The NELF was collected by nasal strips from Leukosorb medium (Pall Corporation). Both the NELF and plasma were aliquoted and stored at -80°C for downstream analysis (details in the supplementary materials). The study was approved by the Joint Chinese University of Hong Kong–New Territories East Cluster Clinical Research Ethics Committee (CREC: 2021.214).

### Measurement of Specific IgA and IgG Against SARS-CoV-2 Spike Protein, SARS-CoV-2 Neutralization Antibody

Semi-quantitative S1-RBD-specific immunoglobulin enzyme-linked immunosorbent assay (ELISA) kits (Euroimmun) were used. Neat NELF and 1:100 diluted plasma were added to the assay wells and processed as per the instructions of the manufacturer. Sample/calibrator (S/C) ratios ≥1.1 were considered as positive, while 15 was the upper detection limit of the assay. A blocking ELISA (GenScript) was employed for SARS-CoV-2 NAb. Thirty percent of signal inhibition cutoff was used to indicate the presence of SARS-CoV-2 NAb in the sample.

### Viral RNA Extraction and Quantification

RNA was extracted from 70 ul of NELF collected at each time point using PHASIFY VIRAL RNA Extraction Kit™, following the instructions of the manufacturer. SARS-CoV-2 was detected by quantitative PCR using the one-step Master Mix (TaqMan Fast Virus, ThermoFisher) with primers targeting the N gene of SARS-CoV-2 (18).

### Statistical Analysis

Subject demographics were compared between the two vaccinated groups using the Mann–Whitney test and Fisher’s exact test as appropriate. For the immunoglobulin profiles, differences between sexes and time points were evaluated using the Mann–Whitney test and Friedman test, followed by Dunn’s multiple-comparison test, respectively.  The correlation of S/C ratio of the specific immunoglobulins with the percentage of signal inhibition in the surrogate neutralization test was examined by Spearman’s correlation test. All statistical tests were performed using Graphpad, version 9.1.2. Differences were considered statistically significant at *p* <0.05.

## Results

### Subject Demographics

Eighty-three subjects were recruited in this longitudinal study. The median age of all subjects was 45 years (range, 21–74); 45% were male. The median age was significantly different in subjects from the two longitudinal vaccine groups, CoronaVac (*n* = 18, median age: 53 years) and Comirnaty (*n* = 65, median age: 42 years) (*p* = 0.0061). All subjects declared no known exposure to SARS-CoV-2-infected subjects. To ensure that the measurements of the change in SARS-CoV-2 specific immunoglobulins were not due to active SARS-CoV-2 infection during the study period, all the NELF samples were also tested for SARS-CoV-2 nucleoprotein gene. In view of the limited number of CoronaVac subjects and the detected difference in age, 15 additional subjects who received CoronaVac were recruited for sampling at the fourth time point, *i*.*e*., any time after 14 days post-booster and before 3 months post-vaccine (**Figure 1B**). Ninety-six subjects, including the longitudinal and cross-sectional groups, completed a questionnaire on local or systemic side effects (see **Supplementary Table S1** in the supplementary materials).

### Comirnaty-Induced Detectable Immunoglobulin in NELF

**Figure 2:**
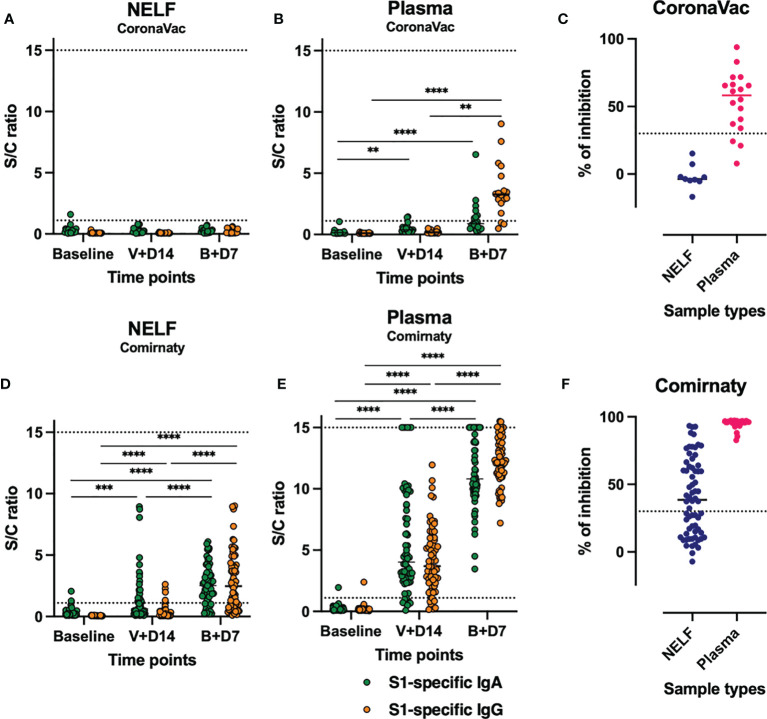
Expression of nasal epithelial lining fluid (NELF) and plasma S1-specific IgA and IgG and neutralizing antibody (NAb). The level of S1-specific IgA (green dots) and IgG (red dots) were plotted against the three standard time points of sample collection in NELF **(A, D)** and plasma **(B, E)** specimens of the recipients of CoronaVac **(A, B)** and Comirnaty **(D, E)**. The data points above the dotted line (sample/calibrator ratio ≥1.1) are considered positive, while the dotted lines at *y* = 15 indicate the upper detection limit of the assay. The asterisks indicate statistical significance between time points of the same Ig class by Friedman test, followed by Dunn’s multiple-comparison test. **p < 0.001, ***p < 0.0005, and ****p < 0.0001. The percentage of signal inhibition observed with the surrogate SARS-Co-V 2 neutralization antibody detection kit by the NELF and plasma samples of CoronaVac **(C)** and Comirnaty **(F)** recipients collected on 7 ± 2 days after the booster is plotted. The 30% signal inhibition cutoff for SARS-CoV-2 NAb detection is interpreted as the sample containing neutralizing antibodies for SARS-CoV-2.

None of the CoronaVac recipients developed detectable NELF S1-specific IgA and IgG (**Figure 2A**) by day 7 ± 2 after the booster. In comparison, most subjects who received Comirnaty developed NELF S1-specific antibodies. The increase in S1-specific IgA and IgG levels at the three time points was significant by Friedman test, followed by Dunn’s multiple-comparison test (**Figure 2D**). Moreover, S1-specific IgA appeared earlier than IgG in NELF. More subjects developed NELF S1-specific IgA (39/65, 40%) than IgG (5/65, 8%) (see **Supplementary Figure E1B**, blue dots, in the supplementary materials) by 14 ± 2 days after the first dose. These further increased to 82 and 68%, respectively, by 7 ± 2 days after the booster (see **Supplementary Figure E1C**, blue dots in the supplementary materials).

### SARS-CoV-2 IgA Appeared Earlier Than IgG in Plasma

In the CoronaVac group, the plasma S1-specific IgA increased significantly by 14 ± 2 days after the first vaccination dose (*p* = 0.0014) and at 7 ± 2 days after the booster (*p* < 0.0001) (**Figure 2B**, green dots). In contrast, a significant increase in S1-specific IgG was detected only at 7 ± 2 days after the booster (*p* < 0.0001) (**Figure 2B**, orange dots) when 83% of the CoronaVac subjects had detectable plasma S1-specific IgG.

In the Comirnaty group (**Figure 2E**), 89 and 91% of the subjects were positive for both plasma S1-specific IgA and IgG by 14 ± 2 days after the first vaccination dose, respectively. By 7 ± 2 days after the booster, all plasma samples were positive for both S1-specific IgA and IgG (see **Supplementary Figure S1C** in the supplementary materials) and neutralizing antibody (**Figure 2E**).

### Negative Correlation Between SARS-CoV-2 S1-Specific Antibody Levels and Age

**Figure 3:**
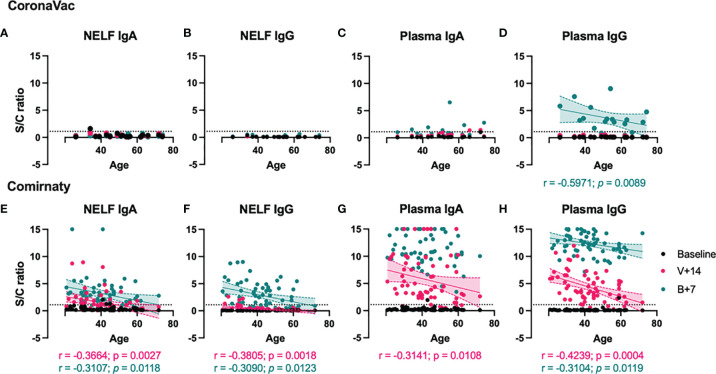
Correlation between S1-specific Igs to the age of the vaccinated subjects. The SARS-CoV-2 S1-specific antibody levels in the nasal epithelial lining fluid (NELF) **(A, B, E, F)** and plasma **(C, D, G, H)** samples detected at baseline, 14 ± 2 days after the first dose (V+14), and 7 ± 2 days after the booster (B+7) time points are plotted against the age of the vaccine recipients. Significant correlations are denoted with the Spearman *r* and the *p*-value. Pink and green dotted lines represent significant linear regression fits with 95% confidence intervals (shaded region with the corresponding colors). Sample/calibrator ≥1.1 is regarded as the threshold of a positive sample, indicated by the horizontal dotted line.

While no S1-specific antibodies were induced in NELF of CoronaVac recipients (**Figures 3A, B**), no correlations analysis could be performed. Moreover, the number of plasma samples with detectable S1-specific IgA level was low and no correlation even at 7 ± 2 days post-booster time point (**Figure 3C**). The induced plasma S1-specific IgG levels were inversely correlated with the age of CoronaVac recipients (*p* = 0.0089, **Figure 3D**). Similar negative correlations between age and induced NELF and plasma antibody were observed in Comirnaty recipients (**Figures 3E–H**), suggesting increased mucosal and serological antibody responses in younger vaccine recipients. Moreover, female subjects (median S/C ratio = 4.845) who received Comirnaty had higher plasma S1-specific IgG at the 14 ± 2-day time point than male subjects (median S/C ratio = 3.020, *p* = 0.0138) (see **Supplementary Figure S2H** in the supplementary materials).

### Neutralization Potential of NELF and Plasma

**Figure 4:**
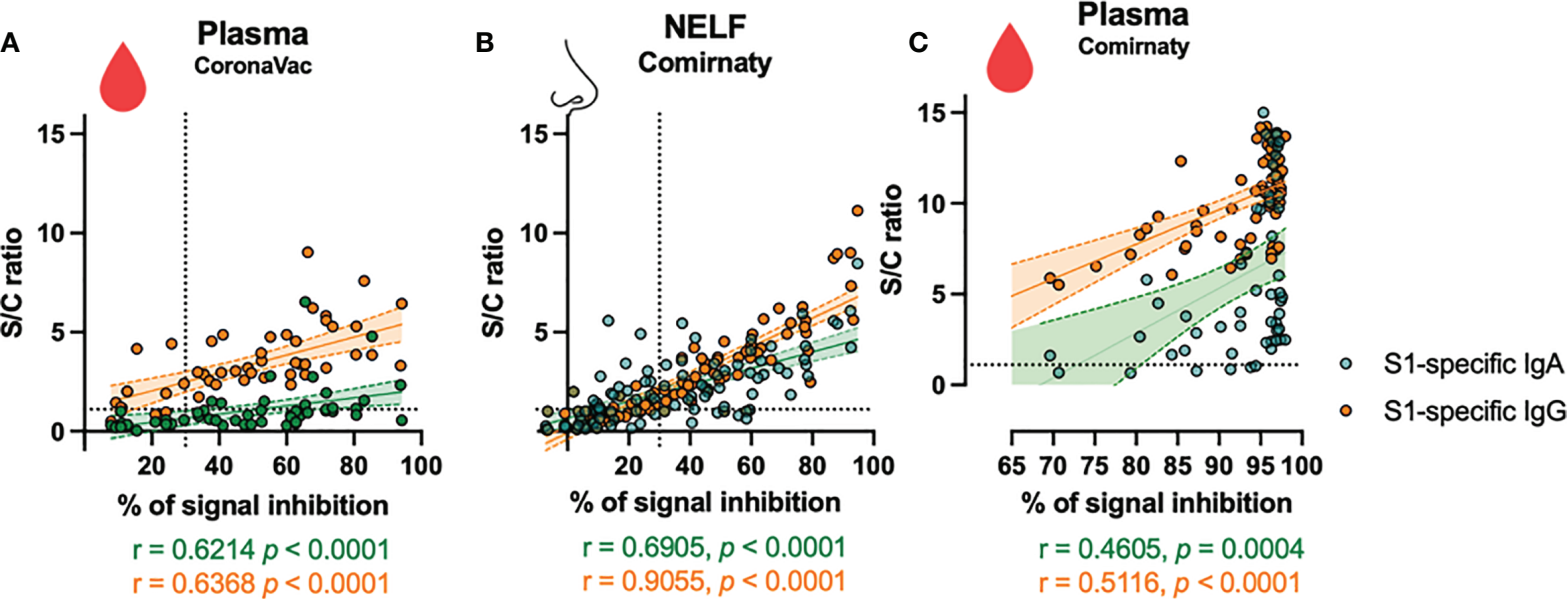
Correlation of S1-specific Igs to the percentage of signal inhibition in the surrogate ACE-2-based neutralization readout. The correlation coefficients of the levels of the **(A)** plasma of CoronaVac subjects, **(B)** nasal epithelial lining fluid (NELF), and **(C)** plasma of Comirnaty subjects at 7 ± 2 days after the booster with the NAb are superimposed on the panel with the trend lines estimated with the use of simple linear regression. The plots show the S/C ratio of the SARS-CoV-2 S1-specific IgA (green dots) and IgG (orange dots) <15 plotted against the percentage of inhibition of the SARS-CoV-2 spike-ACE-2 binding signal, in which inhibition ≥30% is regarded as the threshold of a positive sample, indicated by the vertical dotted line. Out-range specific antibody levels were excluded from the two-tailed Spearman correlation analysis. The green and orange dotted lines represent significant linear regression fits with 95% confidence intervals (shaded region with the corresponding colors).

**Figure 5:**
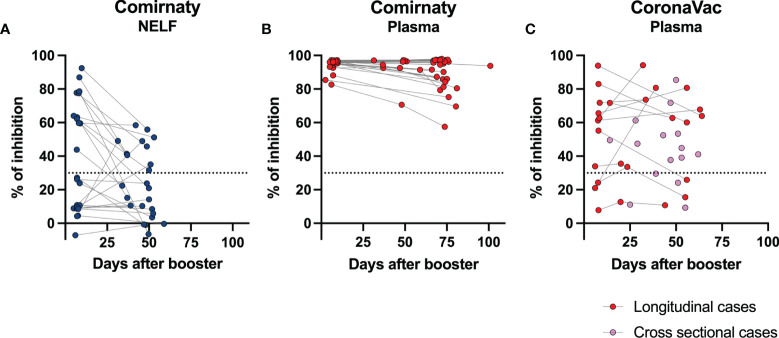
Longevity of the NAb in nasal epithelial lining fluid (NELF) and plasma samples. The paired percentage of signal inhibition in the **(A)** NELF and **(B)** plasma of 24 Comirnaty subjects and **(C)** plasma of CoronaVac recipients in the longitudinal group (*n* = 12, red dots) and in the cross-sectional group (*n* = 15) are shown. The data points of the same individual are joined by a dotted line.

In the CoronaVac group, no NAbs were detected in the NELF samples, which reflected the absence of S1-specific IgA and IgG. Nevertheless, the plasma of 15/18 CoronaVac recipients contained SARS-CoV-2 NAb (**Figure 2C**), and significant correlations were found between the plasma IgA and IgG levels with the percentage of binding inhibition (**Figure 4A**). Twelve subjects provided paired plasma samples collected at 7 ± 2 days post-booster and the fourth sampling time point. Nine remained NAb-positive, and two who were previously negative became positive by 24 days after the booster, while one remained NAb-negative on the 19th and 44 day post-booster (**Figure 5C**, red dots). In the cross-sectional group of the younger CoronaVac recipients, we found that 12/15 plasma samples were neutralizing (**Figure 5C**, pink dots). No correlations were found between NAb levels either with time post-booster (*p* = 0.14) or with age (*p* = 0.28). Overall, 75% (21/28) of the CoronaVac recipients had NAb in their plasma at the fourth sampling time point.

In the Comirnaty group, 37/65 NELF samples inhibited the binding of SARS-CoV-2 spike protein to ACE-2 (**Figure 2F**), whereas all plasma samples were neutralizing and provided over 80% inhibition to the binding of SARS-CoV-2 spike protein to ACE-2. Significant correlations were found between NELF IgA (*r* = 0.6905, *p* < 0.0001), NELF IgG (*r* = 0.9055, *p* < 0.0001) (**Figure 4B**), plasma IgA (*r* = 0.4605, *p* = 0.0004), and plasma IgG (*r* = 0.5116, *p* < 0.0001) (**Figure 4C**) levels and the percentage of binding inhibition. All subjects had plasma NAb since 7 ± 2 days after the booster, and it lasted for at least 50 days after the booster (**Figure 5B**).

### Neutralizing Antibody in NELF Is Transient

The longevity of the NAb in NELF was further assessed in 24/65 Comirnaty subjects who had reached the fourth sampling time point (**Figure 5A**). Eleven out of 24 NELF contained NAb on 7 ± 2 days after the booster, while seven of them remained positive by the fourth sampling time point, and four of them became Nab-negative. Nevertheless, a late NELF NAb development was observed in three individuals who did not possess NELF NAb earlier on at 7 ± 2 days after the booster, though the NELF of 10 subjects remained negative for NAb. Within these 24 subjects, a significant decrease in NAb (*p* = 0.0291) was also observed from 7 ± 2 days after the booster to the fourth sampling time point and in the S1-specific IgA level (*p* < 0.0001) (see **Supplementary Figure S3** in the supplementary materials).

## Discussion

Our study reveals that both Comirnaty and CoronaVac induce plasma SARS-CoV-2 S1-specific IgA, IgG, and NAb. However, Comirnaty, but not CoronaVac, was also able to induce S1-specific IgA and IgG in the nasal mucosa. Our results show that NELF S1-specific IgA was detected earlier compared with S1-IgG, together with a higher proportion of Comirnaty recipients eliciting S1-specific IgA response (82%) than S1-specific IgG response (68%) by 7 days after the booster. Overall, 57% of the NELF samples exhibited a neutralizing activity, and the NAb correlates positively with the levels of S1-specific IgA and IgG. The induction of nasal S1-specific immunoglobulins and NAb is unique to subjects receiving Comirnaty, and it was not found in the NELF of CoronaVac recipients. The longevity of the NAb in NELF was assessed in 24/65 Comirnaty subjects who had reached the extended time point. Furthermore, 64% (7/11) of NELFs remained neutralizing, while the rest (4/11) became NAb-negative. Nevertheless, a late rise in NELF NAb was observed in three individuals who did not possess NAb in the earlier time points. Lastly, 10 of the Comirnaty recipients remained NELF NAb-negative through all time points.

It is commonly believed that intramuscular vaccines do not induce mucosal immunity effectively (19). The mucosal immunity of the upper respiratory tract is partly compartmentalized and usually initiated in the nasopharynx-associated lymphoid tissue (NALT) in all age groups and bronchus-associated lymphoid tissue (BALT) in children and adolescents or adults upon disease induction (20). These upper respiratory tract-associated lymphoid tissues generate IgA-producing mucosal B cells that express the homing receptor, e.g., a4ß1, CCR10, CD62L, and LFA-1 (21, 22). These homing receptors allow the B-cells to traffic efficiently to the mucosal effector site, the respiratory tract in this case, where their ligands, VCAM-1 and CCL28, are strongly expressed. The IgA-producing mucosal B cells differentiate into polymeric IgA-secretory plasma cells and contribute to the production of the polymeric IgA (in dimers or tetramers) in the lamina propria as opposed to serological IgA (predominantly monomers), which is produced within the bone marrow, spleen, and lymph nodes (23). The dimeric IgA is formed by linked two IgA molecules by a joining chain (J-chain), while the J-chain binds to the polymeric immunoglobulin receptor (pIgR), which transports the dimeric IgA from the basolateral to the apical surface of the epithelium by transcytosis. Therefore, the SIgA present in secretions is typically produced within mucosal tissues. This raises important questions about the route that mRNA lipid nanoparticles need to take from the intramuscular injection site to the NALT (and BALT) and the biological mechanisms that underlie this process.

An *in vivo* investigation in the biodistribution of the lipid nanoparticles carrying influenza virus mRNA found that, after intramuscular administration, the concentration of mRNA lipid nanoparticles decreases along the disseminating route from the injection site. The expression of mRNA can be detected in distal tissues, including the lung, though the concentration was 1,000-fold lower (24). We postulate that the number of mRNA lipid nanoparticles that reach the nasal mucosa after Comirnaty injection might be sufficient for NALT stimulation. However, the mechanisms underlying this process and the factors that affect the consistency of this effect require further investigation.

The clinical implication of the induction of nasal SARS-CoV-2 NAb is an increased likelihood of immediate protection at the target site of viral infection. The role that this mucosal immune response may play in reducing the risk of virus transmission should also be considered. However, a rapid decline of the NELF antibody levels was observed around 40 to 60 days after the booster dose. Currently, this study is one of the few pioneer studies investigating the mucosal antibody induction after SARS-CoV-2 vaccinations (25), while our study uniquely monitors the mucosal antibody kinetics with a systematical study design. Data on the longevity of the mucosal antibody and the factors affecting its longevity after mRNA vaccines are still lacking in the field, which warrants further investigation. We postulated that the decline of the NELF antibody might partly be related to the intensity and the duration of the upregulated expression of J-chain and pIgR, which influence the production of dimeric IgA and the active transcytosis of the dimeric IgA to the nasal epithelial lining or the adhesion molecules and chemokine at the mucosal site, which alternate the homing processing of the plasma cell.

The delayed NAb response found in the plasma of CoronaVac recipients compared with those in the Comirnaty group was not surprising. It was reported that the seroconversion rates were 47.8 and 95.6% for S1-RBD-specific IgG for CoronaVac at 14 and 28 days after the booster, respectively (8). Thus, our interim report at 7 ± 2 days post-booster may not have demonstrated the full immune responses elicited by CoronaVac. Nevertheless, there were five CoronaVac recipients who did not develop NAb in their plasma samples even by the fourth sampling time point, in which one of them never exhibited plasma NAb even at 19 and 55 days post-booster. In addition, two CoronaVac recipients from the longitudinal group had their NAb weaned on 55 and 56 days post-booster.

Consistent with our findings, Danese et al. demonstrated that all SARS-CoV-2 antibodies (IgM, IgG, and IgA) begin to rise from 7 to 11 days after the primary dose of Comirnaty, and they also showed that the booster of Comirnaty further increases the levels of IgG against S1/S2 and RBD (13). Both plasma IgG and IgA levels have been found to remain elevated for up to 65 days after the first vaccine dose (13), while Wang et al. reported that, after receiving two doses of mRNA vaccines (Comirnaty or Moderna), high levels of IgM and IgG against S and RBD of SARS-CoV-2 are detectable for up to 8 weeks after the booster (12). Furthermore, our study demonstrated the correlation between plasma IgA levels with the percentage of virus receptor binding inhibition as reported in a previous study (14). Our findings together confirm the reliability of Comirnaty in generating robust humoral immune responses in vaccinated subjects.

The presence of nasal mucosal immunoglobulins after vaccination against COVID-19 has not been previously reported, while we currently have some insights into the durability of serological IgA and IgG response after Comirnaty vaccination. There is currently no information on the longitudinal expression of the immunoglobulins in NELF samples representing mucosal immunity in COVID-19 patients nor in recipients of SARS-CoV-2 vaccine. We are now continuously following these subjects and collecting paired NELF and blood samples at 3 and 6 months after the first dose of vaccination to better understand the longevity of the mucosal immunity elicited by intramuscular vaccination. Such findings could have implications on public health strategies and screening for immunity to enable the resumption of normalcy on a global scale. The insights gained from the different immune profiles between inactivated viral vaccines and mRNA vaccines could help governments optimize public health strategies. While we found that both Comirnaty and CoronaVac induced systemic humoral responses, Comirnaty likely provides enhanced mucosal level immune protection, which we postulate could contribute to the reduction in asymptomatic transmission risk. This may suggest that Comirnaty could be more suitable for individuals who are often in close contact with vulnerable and/or unvaccinated individuals (e.g., old age home workers, pediatricians, and school teachers).

CoronaVac, on the other hand, induced a satisfactory systemic humoral response with neutralizing capacity in most individuals, and there is less concern about the potential for unintended inflammatory or immune reactions in organs/tissues distal to the vaccination site. Being much easier to store and distribute and producing fewer potential unanticipated tissue responses compared with mRNA vaccines, inactivated virus vaccines may be more suitable for large groups of vulnerable populations within the community.

The unexpected mucosal response in mRNA vaccine recipients raises the concern about which other organs/tissues may be affected and whether such reactions may cause unintended side effects with adverse outcomes. Our study, therefore, highlights the necessity of further studies to determine the distribution of mRNA lipid nanoparticles in humans.

The current study has the following limitations:. First, the smaller sample size and the higher median age of the recipients of CoronaVac in the longitudinal group can be argued to have contributed towards the absence in NELF response and a slower and milder plasma response when compared to Comirnaty. However, we recruited cross-sectional subjects to enrich our data for this important early report and demonstrated that age alone could not explain the different immune responses between the vaccines. Second, the NAb measured in this study is a surrogate measure that is solely based on the inhibition of the binding between the SARS-CoV-2 antibody-mediated blockage of ACE2-spike (RBD) protein–protein interaction (26). The protective effects of the intracellular action of NELF IgA in the Comirnaty recipients or the plasma immunoglobulin specific to other SARS-CoV-2 proteins that theoretically could be present in CoronaVac recipients were not considered. Third, we observed tremendous individual variations—for example, some recipients of Comirnaty were found to be negative for S1-specific IgA, IgG, or both IgA and IgG. These variations require a larger sample size to further clarify. Nevertheless, our current study clearly shows qualitative and significant differences in mucosal response between different vaccine technologies.

## Conclusion

Despite being a vaccine administered *via* the intramuscular route, Comirnaty, and likely other mRNA vaccines, induces S1-specific IgA and IgG in the nasal mucosa of vaccine recipients as early as 14 days after the first dose. The NELF neutralizing effect infers protection from SARS-CoV-2 infection at the upper respiratory epithelium when the level of NAb is sufficiently high. This extra arm of protection at the mucosa, on top of the well-characterized serological antibody development, might further reduce the chance of SARS-CoV-2 infection, in addition to its effectiveness in protecting the recipient from hospitalization and severe disease. Though the response may be transient, it is possible that a more rapid elevation in antibodies may occur within the mucosa when the subject is exposed to live viruses, thus conferring protection from infection even before the virus breaches the mucosa. CoronaVac vaccine induces an IgG-dominant response in the plasma of recipients with neutralizing effect but does not produce any mucosal antibody response. The additional information relating to mucosal response and the direct comparison between two vaccine technologies provides critical insights into how to best utilize these different vaccines from a public health point of view.

## Data Availability Statement

The raw data supporting the conclusions of this article will be made available by the authors, without undue reservation.

## Ethics Statement

The study was approved by the Joint Chinese University of Hong Kong–New Territories East Cluster Clinical Research Ethics Committee (CREC: 2021.214). The participants provided their written informed consent to participate in this study.

## Author Contributions

KYC, HL, and RC contributed to conceptualization. SL, JT, KYC, HL, and RC contributed to methodology. SL, JT, KCC, KYC, GF, HL, and RC contributed to investigation. SL, JC, JT, KYC, and RC contributed to visualization. HL, AL, and RC contributed to funding acquisition. SL, JT, KYC, and RC contributed to project administration. KYC, HL, AL, and RC contributed to supervision. SL, JC, and RC contributed to writing—original draft. KCC, KYC, HL, AL, and RC contributed to writing—review and editing. All authors contributed to the article and approved the submitted version.

## Funding

This study received support from the Innovation and Technology Fund (PRP/039/21FX to RC), the Health and Medical Research Fund commissioned grants (COVID190112 to RC), the Chinese University of Hong Kong Direct Grant for Research (2020.075 to RC), and the Hong Kong Institute of Allergy Research Grant 2020 to RC.

## Conflict of Interest

The authors declare that the research was conducted in the absence of any commercial or financial relationships that could be construed as a potential conflict of interest.

## Publisher’s Note

All claims expressed in this article are solely those of the authors and do not necessarily represent those of their affiliated organizations, or those of the publisher, the editors and the reviewers. Any product that may be evaluated in this article, or claim that may be made by its manufacturer, is not guaranteed or endorsed by the publisher.
