# Supplementary material for: The Mucosal and Serological Immune Responses to the Novel Coronavirus (SARS-CoV-2) Vaccines
Source: Front Immunol. 2021 Oct 12;12:744887. doi: 10.3389/fimmu.2021.744887 (PMC8547269; doi:10.3389/fimmu.2021.744887)
Supplement: Supplementary file 1 [file DataSheet_1.docx]

Supplementary Material

The mucosal and serological immune responses to the Novel Coronavirus (SARS-CoV-2) vaccines

Renee WY Chan^1,2,3,4*^, Shaojun Liu^1,2,3,4^, Jonathan Y Cheung^1,2,3,4^, Joseph GS Tsun^1,2,3,4^, Kate C Chan^1,2^, Kathy YY Chan^1,2^, Genevieve PG Fung^1^, Albert M Li^1,2,4^, Hugh Simon Lam^1,2*^

^1^ Department of Paediatrics, Faculty of Medicine, The Chinese University of Hong Kong, Hong Kong SAR.

^2^ Laboratory for Paediatric Respiratory Research, Li Ka Shing Institute of Health Sciences, Faculty of Medicine, The Chinese University of Hong Kong, Hong Kong SAR.

^3^ CUHK-UMCU Joint Research Laboratory of Respiratory Virus & Immunobiology, Department of Paediatrics, Faculty of Medicine, The Chinese University of Hong Kong, Hong Kong SAR.

^4^ Hong Kong Hub of Paediatric Excellence, The Chinese University of Hong Kong, Hong Kong SAR.

*** Correspondence:**Renee WY Chan
[reneewy@cuhk.edu.hk](mailto:reneewy@cuhk.edu.hk)

Hugh Simon Lam
hshslam@cuhk.edu.hk

**Supplementary Method**

*Subject recruitment*

We released the information of this study through the department website, department social media, The Chinese University of Hong Kong (CUHK) mass mail system, CUHK-online form and word of mouth to reach potential subjects and arrange the sampling logistics. Subject who had arranged their own COVID-19 vaccinations with known schedules of vaccine doses were recruited. In Hong Kong, two doses of the SARS-CoV-2 vaccine will be administered intramuscularly. For CoronaVac, the two doses are twenty-eight days apart while for Comirnaty, the two doses are usually twenty-one days apart. All subjects were requested to complete a one-page questionnaire to capture their demographics, past medical history, drug use and the reporting of any adverse effects after vaccination or respiratory tract infections within the study period. Consent was obtained from the participants and the study was approved by the Joint Chinese University of Hong Kong – New Territories East Cluster Clinical Research Ethics Committee (CREC: 2021.214).

*Clinical sample collection regime*

Nasal epithelial lining fluid (NELF) from both nares and 3mL peripheral blood were collected from the subjects at four time points. A pre-vaccination sample pair was collected during the 48-hour period before the day of vaccination. Two post-vaccination sample pairs were collected at 14 ± 2 days after first dose and 7 ± 2 days post-booster as in **Figure 1A**. We confirmed that all samples obtained on 14 ± 2 days after first dose were collected prior to the second dose of vaccine in all cases. An extended sampling timepoint (4^th^ sampling) of biological sample collection was performed any day between 14±2 days after the booster and before 3 months after the first vaccination dose, to assess the intermediate longevity of SARS-CoV-2 specific Ig and NAb responses.

*NELF collection by nasal strips*

Strips were cut from sheets of Leukosorb medium (Pall Corporation, BSP0669) using a laser cutter (CMA960, Department of Biomedical Engineering, CUHK) to the dimensions of 4 mm wide and 40mm long with a marking at 12mm as previously described (1, 2). 100uL of sterile saline were instilled in each nostril of the subject. Strips were inserted into the anterior part of the inferior nasal turbinate of each nostril until the indicator mark was at or close to the base of each naris. After insertion, the nose was pinched for 1 minute to allow thorough absorption of NELF by the strip. Strips were removed and eluted within 24h after collection. To elute NELF, strips were soaked in 300uL PBS on ice for 5 min with a quick vortex. The solution and the strip were transferred to a Costar Spin-X (CLS9301) and centrifuged twice at 13,000 rpm for 2 min at 4°C to elute the NELF from the strip into the 1.5-ml tube. The NELF was aliquoted into small volume vials for downstream analysis of SARS-CoV-2 specific Ig panels and neutralization test and were stored at −20°C until analysis.

*Plasma preparation*

Blood was collected aseptically by venepuncture and transferred into EDTA blood tube. Plasma samples were separated by centrifugation at 4°C, 2000g for 20 minutes, aliquoted into small volume specimens and stored at –80° C until analysis.

*Measurement of specific IgA and IgG* *against SARS-CoV-2 Spike protein*

Semi-quantitative measurements of SARS-CoV-2 Spike protein (S1 domain) specific Ig ELISA Kits (Euroimmun, EI 2606-9601 A and EI 2606-9601 G) were used. Neat NELF and 1:100 diluted plasma were added to the assay well and processed as per manufacturer’s instructions. After subsequent wash and incubation steps with conjugates or substrates, the plates were analyzed according to the manufacturer’s instructions on the Synergy HTX Multi-Mode Reader. Semi-quantitative readout as a ratio between the sample and the calibrator optical density (OD) values was used. The performance was checked by keeping the optical density of the calibrator within the reference value, and the ratio between the positive and negative controls between 1.6-4.2 and 0-0.7, respectively.

*Measurement of SARS-CoV-2 neutralization antibody*

A blocking enzyme-linked immunosorbent assay (GenScript, L00847) was employed. Briefly, NELF, plasma samples and controls were 1:9 diluted and mixed with HRP-RBD solution and incubated at 37°C for 30 minutes. The mixture was then added to the human ACE-precoated plate and incubated at 37°C for 15 minutes and processed as per manufacturer’s instructions. The performance was checked by ensuring that the OD450 must fall below 0.3 for the positive control and above 1.0 for the negative control. A 30% signal inhibition was set as the cutoff for SARS-CoV-2 NAb detection.

*Viral RNA extraction and quantification*

To eliminate the possibility of active SARS-CoV-2 infection during the study period, 70uL of NELF collected at each timepoint were extracted using PHASIFY VIRAL RNA Extraction Kit™ following manufacturer’s instruction. RNA was reconstituted in 20uL of RNase-free water. 4uL of the RNA sample was used in each reaction, and the SARS-CoV-2 RNA was quantified by one-step Master Mix (TaqMan Fast Virus, ThermoFisher) with primers and probe targeting the N gene of SARS-CoV-2 as described (3). Duplicate reaction was conducted on QuantStudio 12K Flex Real‐Time PCR System (Applied Biosystems, Foster City, CA, USA) at the following cycling conditions: reverse transcription at 50°C for 5 min, inactivation of reverse transcriptase at 95°C for 20 s, 40 cycles of PCR amplification (Denaturing at 95°C for 5 s; Annealing/ Extending at 60°C for 30 s). No template control and positive control using cell lysate from SARS-CoV-2 infected human respiratory cells were included in each run.

**Supplementary Results**

**Local and systemic events after vaccination**

In the CoronaVac group (n=33), fifteen subjects were from the cross-sectional group, and eighteen were from the longitudinal group. All of them received two doses of the vaccine. Thirty-one recipients filled in a questionnaire to report their past medical history, local and systemic events after vaccination (**Supplementary** **Table 1A**). Five subjects reported allergic disease (16%). Five subjects had a history of chronic diseases, including hypertension (n=3), hyperlipidemia(n=1), kidney disease(n=1). Four of five were on drug treatment.

In the Comirnaty group (n=65), 125 completed questionnaires were received from sixty-five subjects, of whom sixty completed after the booster and sixty-five completed after the first dose. From the sixty-five subjects who have received both doses of Comirnaty, twenty-two reported history of allergic diseases, including allergic rhinitis (27%), asthma (3%), drug allergy (5%), eczema (12%), and food allergy (10%). Nine subjects reported chronic conditions, including hyperlipidaemia (n=3), hypertension (n=4), and diabetes (n=2). One subject reported autoimmunity disease under the treatment of immunosuppressant.

**Comparing side effects between CoronaVac and Cormiraty after second dose of vaccine**

32% (10/31) CoronaVac recipients experienced injection site pain and a few of them reported systematic side effects including dizziness (n=1), fatigue (n=4), headache (n=1) and insomnia (n=1). A significantly higher incident of local events, including injection site pain (*p*<0.0001), redness (*p*=0.0160) and swelling (*p*<0.0001); and more systemic side effects, including fatigue (*p*=0.0003), fever (*p*=0.0019), headache (*p*=0.0006) and muscle pain (*p*<0.0001) were found in Comirnaty recipients than CoronaVac recipients (**Supplementary Table 1A**).

**Comparing effects after first and second dose of Comirnaty**

The local and systemic side effect between the first and second dose of Comirnaty were comparable. The common local events included injection site pain (83% vs 87%), redness (12% vs 23%) and swelling (20% vs 35%). The common systemic side effect included fatigue (45% vs 52%), fever (12% vs 23%) and muscle pain (37% vs 38%) while more headache (14% vs 35%, *p* = 0.0067) was detected after booster (**Supplementary Table 1B**).

**Reference**

1. Chan RWY, Chan KC, Chan KYY, Lui GCY, Tsun JGS, Wong RYK, et al. SARS-CoV-2 detection by nasal strips: A superior tool for surveillance of paediatric population. J Infect. 2021;82(4):84-123.

2. Rebuli ME, Speen AM, Clapp PW, Jaspers I. Novel applications for a noninvasive sampling method of the nasal mucosa. Am J Physiol Lung Cell Mol Physiol. 2017;312(2):L288-L96.

3. Chu DKW, Pan Y, Cheng SMS, Hui KPY, Krishnan P, Liu Y, et al. Molecular Diagnosis of a Novel Coronavirus (2019-nCoV) Causing an Outbreak of Pneumonia. Clin Chem. 2020;66(4):549-55.

|  | **CoronaVac** | | **Comirnaty** | | ***P* value** |
| --- | --- | --- | --- | --- | --- |
| **Subjects** | N=31 | | N=60 | |  |
| **Age** (median and range) | 37.0 (21.0-74.0) | | 43.0 (20.8-72.0) | | 0.2486 |
| **Gender (male:female)** | 11:20 | | 28:32 | | 0.3741 |
| **BMI** | 20.9 (16.3-27.1) | | 22.5 (16.6-30.4) | | **0.0046** |
| **Allergic disease** |  |  |  |  |  |
| Allergic rhinitis | 4 | 13% | 16 | 27% | 0.1836 |
| Asthma | 0 | 0% | 2 | 3% | 0.5458 |
| Drug allergy | 0 | 0% | 3 | 5% | 0.5483 |
| Eczema | 2 | 6% | 7 | 12% | 0.7129 |
| Food allergy | 1 | 3% | 6 | 10% | 0.4154 |
| **Other disease** |  |  |  |  |  |
| autoimmunity | 0 | 0% | 1 | 2% | >0.9999 |
| diabetes | 0 | 0% | 2 | 3% | 0.5458 |
| hyperlipidemia | 2 | 6% | 3 | 5% | >0.9999 |
| hypertension | 3 | 10% | 4 | 7% | 0.6862 |
| kidney disease | 1 | 3% | 0 | 0% | 0.3407 |
| **Local event** |  |  |  |  |  |
| injection site itch | 1 | 3% | 9 | 15% | 0.1553 |
| injection site pain | 10 | 32% | 52 | 87% | **<0.0001** |
| injection site redness | 1 | 3% | 14 | 23% | **0.0160** |
| injection site swelling | 0 | 0% | 21 | 35% | **<0.0001** |
| **Systemic side effect** |  |  |  |  |  |
| chill | 0 | 0% | 1 | 2% | >0.9999 |
| dizziness | 1 | 3% | 5 | 8% | 0.6598 |
| drowsy | 0 | 0% | 3 | 5% | 0.5483 |
| fatigue | 4 | 13% | 31 | 52% | **0.0003** |
| fever | 0 | 0% | 14 | 23% | **0.0019** |
| headache | 1 | 3% | 21 | 35% | **0.0006** |
| insomnia | 1 | 3% | 3 | 5% | >0.9999 |
| limb pain | 0 | 0% | 5 | 8% | 0.1617 |
| loss of appetite | 0 | 0% | 5 | 8% | 0.1617 |
| muscle pain | 0 | 0% | 23 | 38% | **<0.0001** |
| nausea | 0 | 0% | 1 | 2% | >0.9999 |
| sore throat | 0 | 0% | 1 | 2% | >0.9999 |
| **Contact with Covid-19 patients** | 0 | 0% | 0 | 0% | - |
| **Positive for SARS-CoV-2 test ever** | 0 | 0% | 0 | 0% | - |

**Supplementary Table 1A. Subject demographics, medical history and the local and systemic side effects reported in the CoronaVac and Comirnaty group after booster.** Mann-Whitney test was performed to determine the differences in the age and BMI while Fisher’s exact test (two-tailed) was performed to determine the difference in the medical history, local and systemic side effect between the two vaccine groups.

|  | **After first dose of Comirnaty (n=65)** | | | | **After booster of Comirnaty (n=60)** | | **P value** |
| --- | --- | --- | --- | --- | --- | --- | --- |
| **Local side effect** |  | |  | |  |  |  |
| injection site itch | 4 | 6% | | 9 | | 15% | 0.1442 |
| injection site pain | 54 | 83% | | 52 | | 87% | 0.6255 |
| injection site redness | 8 | 12% | | 14 | | 23% | 0.1576 |
| injection site swelling | 13 | 20% | | 21 | | 35% | 0.0717 |
| **Systemic side effect** |  |  | |  | |  |  |
| chill | 1 | 2% | | 1 | | 2% | >0.9999 |
| diarrhea | 2 | 3% | | 1 | | 2% | 0.2590 |
| dizziness | 1 | 2% | | 5 | | 8% | 0.1040 |
| drowsy | 2 | 3% | | 3 | | 5% | 0.6703 |
| fatigue | 29 | 45% | | 31 | | 52% | 0.4763 |
| fever | 8 | 12% | | 14 | | 23% | 0.1576 |
| headache | 9 | 14% | | 21 | | 35% | **0.0067** |
| limb numbness | 1 | 2% | | 1 | | 2% | >0.9999 |
| limb pain | 1 | 2% | | 5 | | 8% | 0.1040 |
| loss of appetite | 2 | 3% | | 5 | | 8% | 0.2590 |
| muscle pain | 24 | 37% | | 23 | | 38% | >0.9999 |
| nausea | 1 | 2% | | 1 | | 2% | >0.9999 |
| sore throat | 1 | 2% | | 1 | | 2% | >0.9999 |

**Supplementary Table 1B. Local and systemic side effects reported in the Comirnaty group after the first dose and booster.**


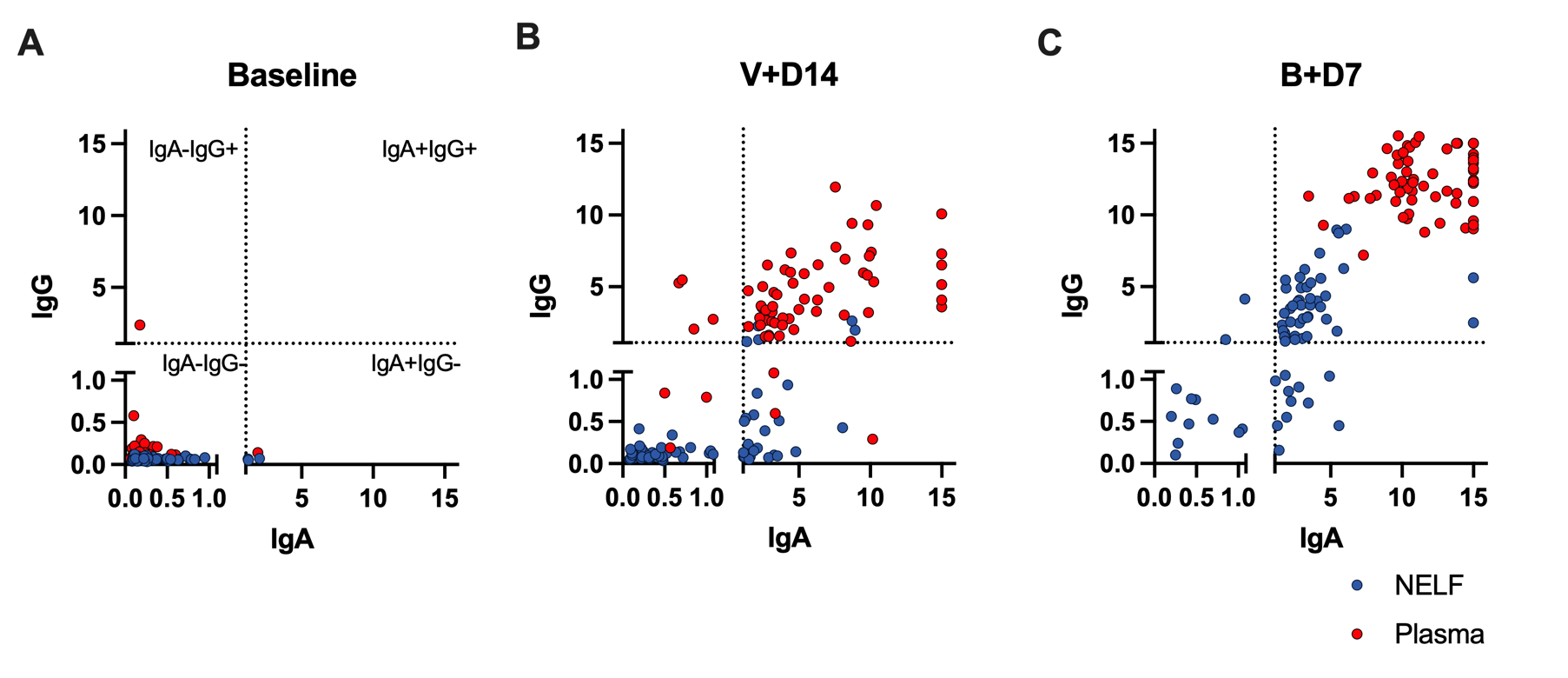


**Supplementary Figure 1.** **Dynamics of IgA and IgG in NELF and plasma of subjects received Comirnaty.** The level of the SARS-CoV-2 S1-specific IgG in NELF (blue dots) and plasma (red dots) were plotted against IgA at **(A)** baseline, **(B)** on 14±2 days after the first dose of Comirnaty and **(C)** 7±2 days after booster. The dotted lines represent the positive thresholds of S1-specific IgA and IgG of the assays, and the dots fall within the four areas represent their nature in having IgA-IgG+, IgA+IgG+, IgA-IG- and IgA+IgG- as illustrated in **(A)**.


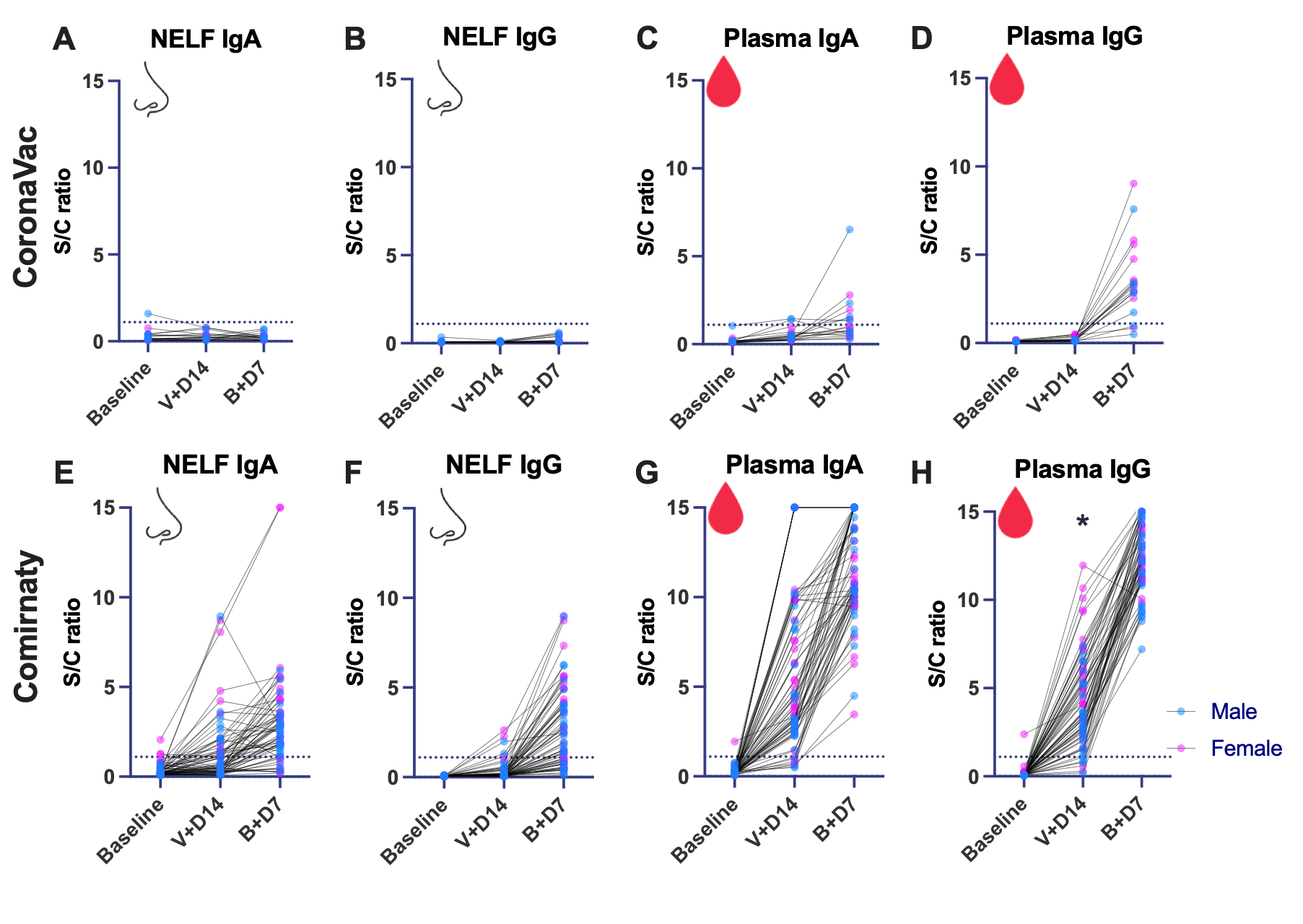


**Supplementary Figure 2. Female recipients of Comirnaty had a higher SARS-CoV-2 S protein specific IgG in plasma on 14±2 days after the first vaccination.** The level of the SARS-CoV-2 S1-specific **(A,D)** IgA in NELF, **(B,E)** IgG in NELF, **(C,F)** IgA in plasma and **(D,G)** IgG in plasma were plotted against the three timepoints of sample collection according to male (n=8; n=29, blue dots) and female (n=10; n=36, pink dots) of the longitudinal **(A-D)** CoronaVac and **(D-G)** Comirnaty group, respectively. The lines connected the Ig levels detected in the same subjects at different time points. Data points above the dotted line (Sample/Calibrator ratio ≥ 1.1) are considered as positive. Asterisk indicates statistical significance between genders at a specific time point (*p* = 0.0138, Mann-Whitney test).


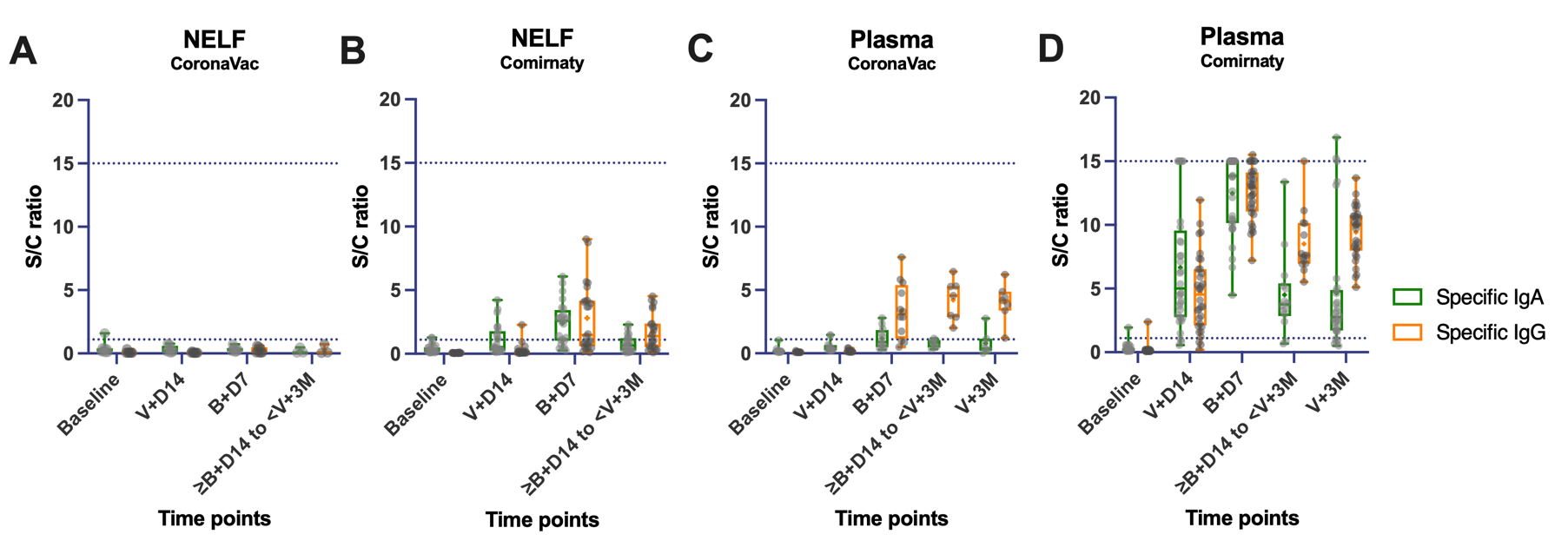


| **E. Comirnaty NELF** | | | | |  | **F. CoronaVac Plasma** | | | | | |
| --- | --- | --- | --- | --- | --- | --- | --- | --- | --- | --- | --- |
|  |  | **Baseline** | **V+D14** | **B+D7** |  |  |  | **Baseline** | **V+D14** | **B+D7** | **≥B+D14 to <V+3M** |
| **S1-IgA** | **V+D14** | 0.0608 | - | - |  | **S1-IgA** | **V+D14** | 0.1600 | - | - | - |
|  | **B+D7** | **<0.0001** | **0.0071** | - |  |  | **B+D7** | **0.0001** | 0.4674 | - | - |
|  | **≥B+D14 to <V+3M** | 0.8766 | >0.9999 | **<0.0001** |  |  | **≥B+D14 to <V+3M** | **0.0117** | >0.9999 | >0.9999 | - |
|  |  |  |  |  |  |  | **V+3M** | 0.2818 | >0.9999 | >0.9999 | >0.9999 |
|  |  |  |  |  |  |  |  |  |  |  |  |
| **S1-IgG** | **V+D14** | 0.1755 | - | - |  | **S1-IgG** | **V+D14** | >0.9999 | - | - | - |
|  | **B+D7** | **<0.0001** | **<0.0001** | - |  |  | **B+D7** | **<0.0001** | **0.0151** | - | - |
|  | **≥B+D14 to <V+3M** | **<0.0001** | **0.0026** | >0.9999 |  |  | **≥B+D14 to <V+3M** | **<0.0001** | **0.0124** | >0.9999 | - |
|  |  |  |  |  |  |  | **V+3M** | **<0.0001** | **0.0149** | >0.9999 | >0.9999 |
|  |  |  |  |  |  |  |  |  |  |  |  |
|  |  |  |  |  |  | **G. Comirnaty Plasma** | | | | | |
|  |  |  |  |  |  |  |  | **Baseline** | **V+D14** | **B+D7** | **≥B+D14 to <V+3M** |
|  |  |  |  |  |  | **S1-IgA** | **V+D14** | **<0.0001** | - | - | - |
|  |  |  |  |  |  |  | **B+D7** | **<0.0001** | **0.0177** | - | - |
|  |  |  |  |  |  |  | **≥B+D14 to <V+3M** | **0.0003** | >0.9999 | **0.0076** | - |
|  |  |  |  |  |  |  | **V+3M** | **<0.0001** | >0.9999 | **<0.0001** | >0.9999 |
|  |  |  |  |  |  |  |  |  |  |  |  |
|  |  |  |  |  |  | **S1-IgG** | **V+D14** | **0.0024** | - | - | - |
|  |  |  |  |  |  |  | **B+D7** | **<0.0001** | **<0.0001** | - | - |
|  |  |  |  |  |  |  | **≥B+D14 to <V+3M** | **<0.0001** | 0.4175 | **0.0471** | - |
|  |  |  |  |  |  |  | **V+3M** | **<0.0001** | **0.0048** | 0.0834 | >0.9999 |
|  |  |  |  |  |  |  |  |  |  |  |  |

**Supplementary Figure 3. Dynamic of SARS-CoV-2 S1-specific IgA and IgG in (A, B) NELF and (C, D) plasma samples in extended time points.** The box and whiskers plots show the median and the min to max value of the S/C ratio of the S1-specific antibodies in 8 subjects received CoronaVac and 24 subjects received Comirnaty at extended time points including baseline, V+D14, B+D7, ≥B+D14 to <V+3M and V+3 month (V+3M). The change in S/C ratio in the NELF of the CoronaVac recipients was not tested as all of them were below the positive threshold level. (**E**) The change in the S/C ratio of the S1-specific IgA and IgG in the NELF of the Comirnaty recipients, the plasma of both the **(F)** CoronaVac and **(G)** Comirnaty recipients were Friedman test. The difference between time points were tested by post hoc Dunn’s multiple comparison test. The *p* values were listed in the corresponding tables.
